# Influence of sociodemographic factors on the health literacy of individuals with mental disorders

**DOI:** 10.1590/0034-7167-2025-0172

**Published:** 2026-06-12

**Authors:** Eliana Elisa Rehfeld Gheno, Marcos Renan Barbosa, Andreia Catia Jorge Silva Costa, Eliane Schmidt, Arnaldo Nogaro, Adriane Cristina Bernat Kolankiewicz

**Affiliations:** I Universidade Regional do Noroeste do Estado do Rio Grande do Sul. Ijuí, Rio Grande do Sul, Brazil; II Universidade de Lisboa. Lisboa, Portugal; III Universidade Regional Integrada do Alto Uruguai e das Missões. Erechim, Rio Grande do Sul, Brazil

**Keywords:** Health Literacy, Mental Disorders, Population Characteristics, Sociodemographic Factors, Education of Patients., Letramento em Saúde, Transtornos Mentais, Características da População, Fatores Sociodemográficos, Educação de Pacientes., Alfabetización en Salud, Trastornos Mentales, Características de la Población, Factores Sociodemográficos, Educación del Paciente.

## Abstract

**Objectives:**

to analyze the influence of sociodemographic characteristics on the health literacy of individuals with severe mental disorders.

**Methods:**

a cross-sectional study was conducted using a sociodemographic questionnaire and the Brazilian version of the Health Literacy Questionnaire (HLQ-Br). The analysis included descriptive and inferential statistics.

**Results:**

married individuals and those whose mothers had up to nine years of education showed greater understanding and support from health professionals. Low parental education was associated with difficulty in obtaining information for health care. Individuals over 54 years old demonstrated greater involvement in active care and better social support. Shorter monitoring time was linked to lower understanding and professional support. Cohabitation favored social support, and higher individual education increased the ability to critically evaluate health information.

**Conclusions:**

health literacy was influenced by age, education level, marital status, duration of follow-up, and household arrangements.

## RESUMO

**Objetivos:**

analisar a influência de características sociodemográficas no letramento em saúde de pessoas com transtornos mentais graves.

**Métodos:**

estudo transversal com aplicação de questionário sociodemográfico e da versão brasileira do *Health Literacy Questionnaire* (HLQ-Br). A análise foi descritiva e inferencial.

**Resultados:**

pessoas casadas e com mães com até 9 anos de escolaridade apresentaram maior compreensão e apoio dos profissionais de saúde. Baixa escolaridade dos pais esteve associada à dificuldade em obter informações para o cuidado com a saúde. Indivíduos com mais de 54 anos demonstraram maior envolvimento em cuidados ativos e melhor suporte social. Menor tempo de monitoramento implicou menor compreensão e apoio profissional. A coabitação favoreceu o suporte social, e maior escolaridade individual aumentou a capacidade de avaliação crítica das informações em saúde.

**Conclusões:**

o letramento em saúde foi influenciado por idade, escolaridade, estado civil, tempo de acompanhamento e arranjos domiciliares

## RESUMEN

**Objetivos:**

analizar la influencia de las características sociodemográficas en el alfabetismo en salud de personas con trastornos mentales graves.

**Métodos:**

estudio transversal con aplicación de un cuestionario sociodemográfico y de la versión brasileña del *Health Literacy Questionnaire* (HLQ-Br). El análisis fue descriptivo e inferencial.

**Resultados:**

las personas casadas y aquellas cuyas madres tenían hasta 9 años de escolaridad presentaron mayor comprensión y apoyo por parte de los profesionales de salud. Un bajo nivel educativo de los padres se asoció con dificultades para obtener información relacionada con el cuidado de la salud. Los individuos mayores de 54 años mostraron mayor participación en cuidados activos y mejor apoyo social. Un menor tiempo de seguimiento se relacionó con una menor comprensión y apoyo profesional. La cohabitación favoreció el apoyo social y un mayor nivel educativo individual incrementó la capacidad de evaluación crítica de la información en salud.

**Conclusiones:**

el alfabetismo en salud estuvo influenciado por la edad, el nivel educativo, el estado civil, el tiempo de seguimiento y los arreglos de vivienda.

## INTRODUCTION

Currently, Health Literacy (HL) has been the focus of research by researchers and its concept has been deepening, based on the understanding of its influence on improving quality and living conditions, autonomy and empowerment of people. At present, the broader concept of HL refers to people’s ability to find, understand, evaluate and use health information and services to promote and maintain health, both individually and collectively. HL develops over time, through lived experiences, social interactions and cultural factors. It is based on inclusive and equitable access, plays a key role in empowering people and the community to make decisions^(1,2)^.

It is considered a multidimensional construct that includes *functional* skills, with which people have reading and writing skills, making it possible to obtain important information for their health; *communicative* skills, where people can find, understand and apply information in making decisions for themselves or helping others; and *critical* skills, in which the person uses information in order to exercise greater control over factors and social determinants that impact health^(3)^.

The Health Literacy Questionnaire (HLQ) is a multidimensional instrument that assesses the potentialities and weaknesses of the HL. It has been increasingly used both for the general population and for those in specific conditions^(4-10)^. Among these, studies that evaluate the HL of people living with mental disorders (MDs) are limited and in the Brazilian scenario were not found.

In Brazil, about 2 million people have MDs and, of these, it is estimated that 3% have severe and persistent MDs, corresponding to 5 million adults. Worldwide, MDs represent about 12% of diseases and result in approximately 1% of mortality rates^(11)^. MDs have resulted in decreased quality of life, functional impairments, greater morbidity and mortality, greater degree of dependence and disability, increased economic costs, producing an impact on the lives of people, families and society, thus being a public health issue of great relevance^(12)^.

Studies evaluating HL levels of people with MDs are scarce, especially using multidimensional instruments. Studies conducted with people with MDs employing the HLQ have been conducted in Australia^(13)^, Denmark^(14)^, Canada^(15)^. Another international study that used the HLQ was carried out with people with substance use disorders^(16)^. In Brazil, no studies were found that evaluated HL from the multidimensional perspective of people with MDs. Based on this context, this study aims to identify the influence of sociodemographic characteristics on the health literacy of people living with mental disorders.

In the Brazilian context, studies have assessed HL in a multidimensional way across different population groups, such as family caregivers^(17)^, healthcare professionals, general users^(6)^, individuals with cardiovascular diseases^(18)^, and hospitalized patients with chronic conditions^(19)^. This study stands out for its unprecedented contribution by employing the HLQ, psychometrically validated in Brazil, to evaluate HL among people with mental disorders^(20)^, with an emphasis on sociodemographic factors, which remain scarcely explored in the national scientific literature.

In this sense, the data generated by this investigation may guide nursing practice in the field of mental health, especially in the development of care management strategies that are more understandable, accessible, and centered on the needs of people with mental disorders. By identifying the levels of HL in this population, professionals will be able to improve communication, enhance educational actions, strengthen the therapeutic bond, and promote greater autonomy and participation of users in decisions regarding their care. Furthermore, the results may support the development of educational materials and institutional policies that are more equitable and inclusive.

## OBJECTIVES

To identify the influence of sociodemographic characteristics on the health literacy of people living with mental disorders.

## METHODS

### Ethical aspects

Study authorized by the authors of the Health Literacy Questionnaire - HLQ by e-mail (hl-info@swin.edu.au). Approved by the Research Ethics Committee under Opinion number 5.966.864/2022. The interviews began after the participants signed the informed consent form.

### Design, data and local of study

Cross-sectional study, the presentation follows the criteria established by the checklist Strengthening the Reporting of Observational Studies in Epidemiology (STROBE), carried out in a Psychosocial Care Center (CAPS) type II, linked to the Unified Health System (SUS), in a municipality with a population of 83,947 inhabitants^(21)^, in Rio Grande do Sul, Brazil. Data collection took place between April and October 2023. The participants were selected by convenience. Use of instruments: Sociodemographic and Health Conditions Questionnaire on age, marital status, cohabitation status, level of education of the individual, as well as the mother and father, length of monitoring in the service; and the Brazilian version of the Health Literacy Questionnaire (HLQ-Br)^(4,6)^. The HLQ-Br has been psychometrically validated for use in people with mental disorders, with αC values of 0.893 and 0.788, respectively, in parts 1 and 2 of the instrument^(20)^.

The HLQ is an instrument to assess people’s comprehension skills and use of health information in a multidimensional way, analyzing the levels of functional, communicative and critical HL^(4)^. It consists of 44 questions, distributed in nine domains, divided into two parts. The first consists of domains 1 to 5, which are scored on a Likert scale of (1) strongly disagree to (4) strongly agree, and the second, domains 6 to 9, scored on the Likert scale of (1) “always difficult” to (5) “always easy”.

The score obtained refers to the potentialities and weaknesses of each domain and the score is the result of the sum of each item of the scales, dividing this total by the number of items on the scale. The result corresponds to the mean score^(4,6)^.

In this manuscript the results of the first part of the HLQ will be presented. We chose to use only the first part of the HLQ, considering the robustness of its scales and their greater relevance for analyzing the relationship between health literacy and sociodemographic characteristics, which was the central objective of this study.

### Sample definition and selection criteria

Data were collected by undergraduate research assistants from Nursing and Medicine programs in a private room to ensure participant privacy. The assistants were trained in advance by the first author and the project coordinator, through review and explanation of each instrument’s items.

Were included people aged 18 years or older, with a medical diagnosis of severe mental disorder recorded in the medical record, psychically stable, which is considered a state of mental and emotional balance that enables the person to adequately cope with the internal and external demands of daily life. Were excluded are those with concomitant medical diagnosis of intellectual or mental disability or retardation, as well as those under judicial interdiction. This study is part of a matrix project that performed sample calculation for psychometric validation of an instrument for use in people with mental disorders (MDs). Sample size estimates of 90% and margin of error of 9.5 were used, from a weighted population of 720 people. The minimum size was 432 people. 512 people were invited and, of these, the response rate was 87%. The administration time of the questionnaires ranged from 15 to 20 minutes.

### Data analysis and treatment

For statistical decision criteria, a significance level of 5% was adopted. In descriptive statistics, absolute (n) and relative (%) distributions were used, as well as measures of central tendency and variability, with the study of normality of data distribution using the Kolmogorov-Smirnov test.

The comparison of the scores of the HLQ scale with the dimensions from D1 to D5 occurred through the Analysis of Variance (One Way) - Post Hoc Bonferroni. And, in situations where the means were compared between two groups, the Student’s t-test was used for independent groups.

## RESULTS

Four hundred and forty-four (444) people with MDs participated. Females predominated (337; 75.9%), who had no partner (258; 58%), although they were living with other people, were children, partners or people with other ties (370; 83.3%). They declared themselves white (340; 76.5%), with a predominant age group between 18 and 54 years (298; 67.1%) and with an income of up to two minimum wages (344; 77.4%).

Participants with education of up to 9 years predominated (256; 57.65%), as well as the education of the father and mother, respectively (211; 47.52% and 250; 56.31%) were higher in this range. Regarding the results of the evaluation of the HLQ instrument, Table 1 presents the comparison of domains D1 - Understanding and support of health professionals and D2 - Sufficient information to take care of health with sociodemographic characteristics and health conditions.

**Table 1:** Mean and standard deviation for domains D1 and D2, according to marital status, monitoring time and parental education, Ijuí, Rio Grande do Sul, Brazil, 2023

	n	D1 - Understanding and support of health professionals		D2 - Sufficient information to take care of health	
		Mean	Standard deviation	Mean	Standard deviation
Marital status					
Not common-law married	258	2.93	0.37	2.97	0.41
Married/common-law married	186	3.45	0.46	2.82	0.43
*p* (value)^B^		0.041		0.611	
Monitoring time:					
Under 6 months	72	2.82	0.40	2.74	0.53
From 6 months to 1 year	41	3.01	0.44	2.94	0.40
From 1 to 2 years	36	3.00	0.29	2.87	0.34
From 2 to 5 Years	80	2.98	0.43	2.86	0.51
Over 5 years	215	3.00	0.41	2.82	0.46
*p* (value)^A^		0.021		0.212	
Mother’s education					
None	55	2.86ab	0.56	2.63b	0.51
1 to 4 years	141	3.03a	0.35	2.88ª	0.45
5 to 9 years	54	3.04a	0.44	2.94ª	0.43
10 to 12 years	37	2.93ab	0.42	2.79ab	0.44
Over 12 years	13	2.85b	0.28	2.73b	0.36
*p* (value)^A^		0.003		0.004	
Father’s Education					
None	57	2.89	0.51	2.66b	0.51
1 to 4 years	117	3.05	0.37	2.93ª	0.44
5 to 9 years	37	2.95	0.26	2.86ª	0.35
10 to 12 years	26	2.89	0.29	2.75ab	0.51
Over 12 years	19	3.08	0.45	2.82ab	0.51
*p* (value)^A^		0.071		0.006	

*A: Analysis of Variance Test (One Way) - Post Hoc Bonferroni, where means followed by equal letters do not differ at a significance of 5%. B: Student’s t-test for independent groups.*

When comparing the D1 domain, we identified a statistically significant difference in marital status (p=0.041). Participants who declared themselves married or in a stable union had a significantly higher mean score compared to the group that reported not living in a union.

The monitoring time stood out over the D1 domain scores (p=0.021), and points out that patients with less than six months of monitoring (2.7±0.2) concentrated lower scores when compared to monitoring periods greater than six months.

Furthermore, when comparing the mother’s education level (p=0.003), the education levels of 1 to 4 years and 5 to 9 years of study presented significantly higher mean scores when compared to mothers with education above 12 years.

In the D2 domain, we identified a significant impact of parental education. As shown in Table 1, at the mother’s education level (p=0.004), the lowest mean scores occurred among those with no education (2.63±0.51) and with education above 12 years (2.73±0.36), compared to education levels from 1 to 4 (2.88±0.45) and from 5 to 9 years (2.94±0.43). As for the paternal education level (p=0.006), again the lowest mean score occurred among uneducated fathers (2.66±0.51), compared to fathers with education levels from 1 to 4 (2.93±0.44) and from 5 to 9 years (2.86±0.35).

In the results of D3 - Active health care, there is evidence of a statistically significant difference in the age group (p=0.008), so that people aged over 54 years (2.79±0.39) had a higher mean score compared to the age groups of 18 and 34 (2.62±0.42) and 35 and 44 years (2.65±0.38), as shown in Table 2.

**Table 2:** Mean and standard deviation for the D3 domain, according to age group and monitoring time, Ijuí, Rio Grande do Sul, Brazil, 2023

	n	D3 - Active health care	
		Mean	Standard deviation
Age group			
From 18 to 34 years	80	2.62b	0.42
From 35 to 44 years	101	2.65b	0.38
From 45 to 54 years	117	2.67ab	0.45
Over 54 years	146	2.79a	0.39
*p* (value)^A^		0.008	
Monitoring time:			
Under 6 months	72	2.57b	0.42
6 months to 1 year	41	2.65b	0.49
From 1 to 2 years	36	2.82a	0.42
From 2 to 5 Years	80	2.76a	0.40
Over 5 years	215	2.70ab	0.39
*p* (value) ^A^		0.015	

*A: Analysis of Variance Test (One Way) - Post Hoc Bonferroni, where means followed by equal letters do not differ at a significance of 5%.*

Regarding the monitoring time, the significant difference was also achieved when comparing the mean scores for the D3 domain (p=0.015), indicating that people with monitoring time less than 6 months (2.57±0.42) and from 6 months to 1 year (2.65±0.49) had significantly lower means when compared to patients with monitoring time of 1 and 2 years (2.82±0.42) and 2 and 5 years (2.76±0.40).

Regarding the characteristics that impacted the scores of the D4 - Social support for health domain, the age group (p=0.045) and the fact of living with other people (p<0.001) stood out. As shown in Table 3, we found that people in the age group above 54 years (2.83±0.46) had a significantly higher mean compared to the age groups 35 to 44 (2.66±0.59) and 45 to 54 years (2.69±0.49).

**Table 3:** Mean and standard deviation for dimension D4, according to age group and cohabitation, Ijuí, Rio Grande do Sul, Brazil, 2023

	n	D4 - Social support for health	
		Mean	Standard deviation
Age group			
From 18 to 34 years	80	2.80ab	0.39
From 35 to 44 years	101	2.66b	0.59
From 45 to 54 years	117	2.69b	0.49
Over 54 years	146	2.83a	0.46
*p* (value)^A^		0.045	
Lives with other people			
Lives alone	74	2.56	0.44
Lives with other people	370	2.79	0.49
*p* (value)^B^		<0.001	

*A: Analysis of Variance Test (One Way) - Post Hoc Bonferroni, where means followed by equal letters do not differ at a significance of 5%. B: Student’s t-test for independent groups.*

Regarding the impact of living or not with other people, we identified that people who reported living alone (2.56±0.44) had a significantly lower mean score compared to the group that confirmed living with other people (2.79±0.49).

Regarding the domain D5 - Evaluation of health information, the only representative result found was in relation to the person’s education (p<0.001), where the group with more than 12 years of study (2.78±0.58) had a significantly higher mean score when compared to the lowest levels of education, represented by education of up to 4 years (2.49±0.47) and 5 to 9 years of study (2.50±0.44), as shown in Table 4.

**Table 4:** Mean and standard deviation for dimension D5, according to the person’s education level, Ijuí, Rio Grande do Sul, Brazil, 2023

	n	D5 - Evaluation of health information	
		Mean	Standard deviation
Schooling			
Up to 4 years	121	2.49b	0.47
From 5 to 9 years	125	2.50b	0.44
From 10 to 12 years	140	2.68ba	0.46
Over 12 years	58	2.78ª	0.58
*p* (value) ^A^		<0.001	

*A: Analysis of Variance Test (One Way) - Post Hoc Bonferroni, where means followed by equal letters do not differ at a significance of 5%.*

## DISCUSSION

The study showed that marital status, duration of follow-up in the service, age, cohabitation, and the educational level of the individual and their parents are associated with the strengths and weaknesses of multidimensional health literacy functional, communicative, and critical when analyzed in relation to the domains of the first part of the HLQ. This study represents an important milestone, as it was conducted in a public mental health outpatient service (CAPS) that provides care for individuals with severe, chronic, and persistent mental disorders.

The results of the study showed potential in the domain *Understanding and support of HL health professionals* in people with MDs, in relation to the characteristics of the participants, that is, the population studied is linked to at least one health professional who knows well and trusts for guidance, advice and relevant information to help them understand the information and make decisions about their health^(4)^.

Regarding the domain *Understanding and support of health professionals,* those married or in a stable union had higher scores. They possibly have greater emotional support and encouragement for the adoption of healthy behaviors, and this facilitates the sharing of information, favors the greater ability to deal with health problems, and promotes more open communication and co-responsibility about care, resulting in better adherence to treatment. Likewise, studies showed that married people, despite not presenting a statistically significant difference, had a greater ability to understand and obtain support from health professionals (communicative literacy), among the research participants studied, both with people with severe MDs^(15)^ and with older people with chronic conditions^(7)^.

Likewise, the mother’s education up to 9 years of study presented higher scores, representing that people with MDs can have a greater understanding of the social, economic, cultural and structural complexities that influence the ability to deal with health and life issues, strengthening greater community ties and social and emotional support networks. In this sense, we can infer that professionals are open to promoting adequate communication to individual needs, valuing the experiences lived, seeking to understand the family dynamics, as well as the support network, and encouraging greater bonding of the people and their families with the service team.

Studies carried out in Australia with people with MDs^(13)^, with people with substance use disorder^(16)^ and with the general population, including people with depression and anxiety^(5)^, showed greater fragility in the understanding and support of health professionals, while another study carried out in Quebec^(15)^ showed potential in this area. However, none of the studies compared with the sociodemographic variables.

Unlike what was evidenced in the previous domain, the lower level of education of the mother and father obtained worse results when compared to the domain *Sufficient information to take care of health*. Other studies showed weaknesses in this area^(4,5,8)^, which represents that there is insufficient knowledge and information to live with and manage health problems^(4)^, but did not include parental education as a dependent variable.

When people value the importance and take responsibility for their own health, acting proactively in relation to their own care and taking responsibility for decisions related to their health, placing health as a priority in their lives, this is considered potential in the communicative and critical HL^(4)^ and represents *active health care*. When we compared this domain with the age group, the present investigation showed that, as age increases, active health care improves, corroborating a study carried out with the general population that showed that advanced age improves HL^(5)^. Another study carried out with people with severe MDs showed greater fragility in the HL, when comparing this domain with the age group of the participants, although there was no statistically significant difference^(15)^.

The shorter monitoring time in the service represents weakness in the understanding and support of health professionals and in active health care, negatively influencing the quality and effectiveness of the care offered to people with MDs. This can be explained by the fact that time allows establishing therapeutic relationships, adequately understanding the needs of individuals, stimulating adherence to treatment, early detection of changes, preventing complications, psychoeducation and integration with other support services. Thus, educating patients about their condition, treatment and self-management depends on longer monitoring of individuals. Studies comparing D1 and D3 with monitoring time were not found.

A study^(16)^ showed that older people have greater social support that provides all the support that the person wants or needs to maintain health^(4,7)^, presenting greater communicative and critical skills for HL. Another study^(5)^ also considered age below or above 65 years for comparison with this domain and people with younger ages had greater social support for health.

Study finds that social support reduces perceived stress and distress, leading to better overall health for people^(22)^. In this sense, it is essential for people to continuously have adequate social support, whenever necessary, for the maintenance of physical and mental health^(22)^. Studies explain the importance of family support, with family involvement in HL among people with substance use disorders^(16)^. A Brazilian study conducted with people living with chronic diseases, aimed at assessing health literacy, found that social support is also associated with low levels of education^(19)^.

Finally, presenting expanded cognitive and social skills contributes to critically analyzing information and, thus, having better conditions to control events and situations experienced, for safe and adequate decision making. Thus, having a better ability to evaluate health information and use it in decision-making was influenced by higher education (p<0.001) in this study, corroborating other studies^(5,7)^. Contrary to these results, a study carried out with people with severe MDs showed that people with a lower level of education had higher scores in the evaluation of health information, although they did not present a statistically significant difference^(15)^.

This study allowed us to understand the relationship of sociodemographic variables and health conditions with the HLQ domains: D1 - Understanding and support of health professionals; D2 - Sufficient information to care for health; D3 - Active health care; D4 - Social support for health; and D5 - Evaluation of health information of people living with MDs. The study confirmed and refuted evidence from previous studies. It had comparisons, for the most part, with studies carried out with the general population or other health conditions, in view of the scarcity of studies with people with MDs.

Significant impact was identified for: D1 and marital status, monitoring time and mother’s education; D2 and mother’s and father’s education; D3 and age group and monitoring time; D4 and age group and living with other people; D5 and education level, whose variables showed potentialities and weaknesses in the HL of this population.

Despite the non-standardization of the sociodemographic characteristics researched, the results of the studies allow us to infer that it is essential to adapt communication to the needs of people with MDs, especially when considering the complexity of the information and the greater cognitive difficulty of this population. In addition, the support offered to people being monitored in the service needs to be strengthened.

### Study limitations

Of the study, we point out that the scarcity of studies relating sociodemographic variables and health conditions, including monitoring time and parents’ level of education, with the HL of people living with MDs made it difficult to discuss and interpret the results.

### Contributions to nursing, health or public policy

The results may contribute to other studies, both in the Brazilian and international contexts, considering the scarcity of studies that relate sociodemographic characteristics and health conditions with the functional, communicative and critical HL of people living with MDs, using a multidimensional HL assessment instrument. Considering the results in the evaluation of the care offered to this population, as well as the management practices of mental health services, may be useful to qualify the HL of people, in addition to resulting in better individual, professional and management health conditions. It is also recommended that future studies incorporate the variables of literacy (reading and writing skills) and time since diagnosis, as these factors may significantly influence the results.

## CONCLUSIONS

This study allowed us to identify the factors that influence HL, which were age, length of education, marital status, length of monitoring and cohabitation.

It made it possible to identify the weaknesses of the HL of people with MDs that need to be improved and potentialities that can support the planning of strategies to qualify the health care of this population. The results highlight the importance of listing intervention strategies based on individual, family and sociocultural needs.

Considering the characteristics that influenced the HL and considering social and demographic diversity, vulnerability and cognitive skills can contribute to promoting equity, improving health outcomes and promoting the HL of people living with MDs. The use of the HLQ - an instrument that allows the evaluation of HL in a multidimensional way, at the functional, communicative and critical levels - was essential so that the results found can serve as a basis for promoting interventions in the practice of health services and in the research to be carried out.

## Data Availability

The research data are available only upon request.
